# Efficacy of Herbal vs. Chlorhexidine Mouthwash in Experimental Gingivitis: A Cross-over Clinical and Microbiological Study

**DOI:** 10.3390/dj13120608

**Published:** 2025-12-18

**Authors:** Zaineb Aslam, Jamie Wu, Zhong Wang, Nina K. Anderson, Nathan E. Estrin, Georgios E. Romanos

**Affiliations:** 1 Department of Periodontics and Endodontics, School of Dental Medicine, Stony Brook University, Stony Brook, NY 11794-8712, USA; z.aslamdds@gmail.com (Z.A.); jamiewu1998@gmail.com (J.W.); zhong.wang@stonybrookmedicine.edu (Z.W.); 2 Department of Oral Biology & Pathology, School of Dental Medicine, Stony Brook University, Stony Brook, NY 11794-8712, USA; nina.anderson@stonybrookmedicine.edu; 3 Department of Periodontics, The University of Iowa College of Dentistry and Dental Clinics, Iowa City, IA 52242, USA; drnathan@estrinperiodontics.com

**Keywords:** chlorhexidine, cross-over study, experimental gingivitis, mouthrinse, oral rinse, periodontal therapy

## Abstract

**Background**: Chlorhexidine (CHX) is an effective antiseptic rinse for managing gingival inflammation; however, side effects such as staining and altered taste limit its long-term use. StellaLife^®^ (SL), an herbal-based mouth rinse and a gel, has shown promising in vitro effects, including enhanced biocompatibility and wound healing. This study aimed to compare the clinical efficacy of SL and 0.12% CHX in an experimental gingivitis model. **Methods**: In this randomized, controlled, cross-over clinical trial, 34 dental students received both treatment regimens in alternating two-week phases following prophylaxis. Group 1 used SL (mouth rinse and the gel) and then crossed over to CHX with placebo gel. Group 2 followed the reverse sequence. Participants refrained from oral hygiene during treatment phases. Clinical parameters and gingival crevicular fluid (GCF) were assessed at baseline and post-treatment. Paired *t*-tests and Bonferroni corrections were applied (*p* < 0.05). Bacterial count was determined by an external laboratory using a PCR test. Mean values for bacteria after SL and CHX use measured in genome copies/mL for *Aggregatibacter actinomycetemcomitans*, *P. gingivalis*, *T. denticola*, *T. forsythia* and *F. nucleatum*. **Results**: No statistically significant differences were observed between the SL and CHX groups for PI (*p* = 0.057), GI (*p* = 0.960), PD (*p* = 0.112), BOP (*p* = 0.895), GR (*p* = 0.768), CAL (*p* = 0.112), or GCF (*p* = 0.951). Both regimens improved periodontal parameters similarly. No significant differences were found between CHX and SL use in respect to periodontal pathogenic bacteria in the oral cavity. **Conclusions**: SL demonstrated clinical efficacy comparable to CHX in managing experimental gingivitis. Given its favorable safety profile, SL may serve as a promising alternative to CHX, though larger and longer-term studies are warranted.

## 1. Introduction

Periodontal diseases are among the most common chronic inflammatory conditions of the oral cavity, with dental plaque playing a central role in their pathogenesis [1,2]. Plaque is a structured microbial biofilm that initiates gingival inflammation and, if uncontrolled, can progress to periodontal destruction [3,4,5,6,7,8]. Traditionally, antiseptic agents such as chlorhexidine (CHX) have been employed to control plaque accumulation and mitigate gingival inflammation, and CHX remains the gold standard antiseptic recommended in most periodontitis treatment guidelines. However, its long-term clinical use is limited by well-documented side effects, including tooth staining and altered taste sensation, which reduce patient acceptance and compliance. These limitations have prompted the search for alternative agents with comparable efficacy but improved tolerability.

Herbal-based oral care products have gained attention as potentially effective and more biocompatible alternatives [9,10]. StellaLife^®^ (SL), available as a rinse and gel, contains natural ingredients such as *Azadirachta indica* (Neem), *Calendula officinalis*, and *Plantago major* that possess antimicrobial, anti-inflammatory, and wound-healing properties [9,11,12,13,14,15,16,17]. Neem provides numerous bioactive compounds, while Calendula and Plantago have also demonstrated antimicrobial and tissue-repair benefits [11,12,13,14,15,16,17].

The superior biocompatibility of SL compared to CHX has been documented in in vitro studies. Fujioka-Kobayashi et al. [18] showed that brief exposure to SL preserved fibroblast viability, whereas CHX significantly reduced survival. Zhou et al. [19] further demonstrated that SL promoted wound closure and collagen deposition, while CHX impaired healing.

Despite these promising laboratory findings, clinical data comparing the efficacy of SL with conventional CHX in managing plaque and gingivitis remain scarce. In addition to clinical outcomes, microbiological assessment targeted five key bacterial species: *Aggregatibacter actinomycetemcomitans*, *Porphyromonas gingivalis*, *Treponema denticola*, *Tannerella forsythia*, and *Fusobacterium nucleatum*. These organisms were selected because they are recognized periodontal pathogens within Socransky’s red and orange complexes and are strongly associated with plaque maturation, gingival inflammation, and periodontal tissue destruction. [20,21]

Although gingivitis typically resolves after prophylaxis and resumption of oral hygiene, the experimental gingivitis model is a well-established research tool for evaluating the antiplaque and anti-gingivitis effects of antiseptic agents under conditions of deliberately suspended oral hygiene. Therefore, this randomized, controlled, cross-over study was conducted to evaluate and compare the short-term clinical and microbiological effects of SL and 0.12% CHX in an experimental gingivitis model.

## 2. Materials and Methods

### 2.1. Patient Selection

Before subject recruitment and data collection began (ClinicalTrials.gov:NCT07270952 2025-12-03), approval was obtained from the Institutional Review Board (IRB) with the study number IRB2022-00048 (Approval date: 24 August 2022). A double-blind, cross-over study was conducted with a total of 34 participants initially recruited from the dental and pre-dental student body. This population was chosen to ensure a similar age range, standardized oral hygiene practices, and a commitment to dental research. Prior to the study, all participants provided informed consent. At the initial screening visit, a comprehensive medical history was reviewed to determine eligibility based on the inclusion and exclusion criteria. Inclusion criteria required subjects to have good oral hygiene without periodontal pockets. Exclusion criteria included known allergies, impaired wound healing, autoimmune diseases, intolerance to herbal components, HIV/AIDS, and sensitivity to mercury compounds. Following screening, 31 participants (ages 21–30) who were periodontally healthy and free of conditions, such as localized periodontitis, rapidly progressive periodontitis, or acute necrotizing ulcerative gingivitis were enrolled.

### 2.2. Study Design

All participants underwent an initial screening, including review of medical and dental records, saliva sampling, and full periodontal charting. Clinical evaluations were performed at baseline, 2 weeks, and 4 weeks, and included probing depth (PD), bleeding on probing (BOP), gingival margin/recession (GM), clinical attachment loss (CAL), gingival crevicular fluid (GCF) volume, and gingival and plaque indices (GI and PI, according to Löe & Silness [22] and Silness & Löe [23]. Although patients with periodontitis were excluded, PD and CAL were recorded systematically as part of periodontal charting. Minor variations in PD (≤3 mm) and CAL may reflect normal sulcus depth or localized recession and do not indicate periodontitis.

This was a randomized, controlled, cross-over design. After baseline examination and professional prophylaxis, participants were randomly assigned to one of two sequences:

**Group A (SL → CHX):**

Phase I (Weeks 0–2): SL mouthwash (3×/day, 1–2 min) + SL gel applied to the gingival margin (1 min with a Q-tip).

Phase II (Weeks 3–4): After re-evaluation and prophylaxis, 0.12% chlorhexidine (CHX, Peridex^®^) mouthwash + placebo gel (vehicle without active ingredients).

**Group B (CHX → SL):**

Phase I (Weeks 0–2): CHX mouthwash + placebo gel (same regimen as above).

Phase II (Weeks 3–4): After re-evaluation and prophylaxis, switched to SL mouthwash + SL gel.

Participants were instructed to refrain from toothbrushing and flossing for the duration of the study and to avoid eating or drinking for 15 min after each product application. At the end of each 2-week phase, clinical assessments and saliva samples were collected. Participants also completed a short survey on product taste, staining, and ease of use.

### 2.3. Clinical Periodontal Parameters

A comprehensive periodontal examination was conducted by a single, calibrated examiner to ensure consistency. The evaluation included measurements in the following order: PD, BOP, GM, CAL, gingival index (GI), and plaque index (PI).

Gingival Index (Loe and Silness, [22]):

0: Absence of inflammation.

1: Mild inflammation—slight change in color and texture.

2: Moderate inflammation—moderate glazing, redness, edema, and hypertrophy; bleeding on probing present.

3: Severe inflammation—marked redness and hypertrophy with a tendency for spontaneous bleeding and ulceration.

Plaque Index (Silness and Loe, [23]):

0: No plaque.

1: A film of plaque adhering to the free gingival margin, detectable only after disclosing or probing.

2: Moderate accumulation of soft deposits within the gingival pocket or on the tooth surface, visible to the naked eye.

3: Abundance of soft matter within the gingival pocket and/or on the tooth surface.

### 2.4. Gingival Crevicular Fluid (GCF) Monitoring

GCF was collected prior to any periodontal probing during the different visits to minimize contamination. Using a Periotron R8000 device (Oraflow Inc., Smithtown, 11787 NY, USA) calibrated with dry filter strips (confirmed with a baseline reading of 0), filter paper strips were placed in the intrasulcular mesial-buccal (MB) region of one maxillary first molar and one mandibular first molar for 15 s. The strips were then immediately placed on the Periotron platform, and the readings were recorded to quantify the GCF volume.

### 2.5. Microbiological Assessment

At baseline, 2-week, and 4-week visits, 2 mL of whole saliva was collected using the passive drool method. Samples were stored in cooled containers and shipped immediately to an external laboratory (Direct Diagnostics, Austin, TX, USA) for blinded diagnostic analysis. At baseline, 2-week, and 4-week visits, 2 mL of whole saliva was collected using the passive drool method. Saliva sampling was chosen because it is non-invasive, easily standardized across participants, and allows repeated longitudinal collection. Although saliva does not fully reflect site-specific plaque biofilm composition, it provides a reproducible overview of oral microbiota and has been shown to correlate with periodontal status in previous studies. Samples were stored in cooled containers and shipped immediately to an external laboratory (Direct Diagnostics, Austin, TX, USA) for blinded diagnostic analysis.

Bacterial counts of *Aggregatibacter actinomycetemcomitans* (A.a.), *Porphyromonas gingivalis*, *Treponema denticola*, *Tannerella forsythia*, and *Fusobacterium nucleatum* were determined using quantitative polymerase chain reaction (qPCR). DNA was extracted from saliva samples using a proprietary magnetic bead-based method validated by the laboratory. Species-specific primers targeting the 16 S rRNA gene regions were used for amplification. Quantification was performed by comparing cycle threshold (Ct) values against standard calibration curves generated from known concentrations of each bacterial species. Final results were expressed as bacterial counts (cells/mL of saliva). All analyses were performed under blinded conditions.

### 2.6. Sample Size Calculation

A priori power analysis was conducted following Cohen [24] to determine the minimum sample size required to test the study hypothesis. The analysis indicated that a sample size of *N* = 26 would provide 80% power to detect a large effect at a significance level of α = 0.05. Accordingly, a final sample size of *N* = 31 was deemed sufficient to adequately test the hypothesis.

### 2.7. Statistical Analysis

Clinical data, including bacterial counts, PD, BOP, GM, GI, PI, CAL, and GCF, were compiled for both groups (totaling 31 participants) at baseline, 2 weeks, and 4 weeks. Mean values and standard deviations were calculated for each parameter. Intergroup comparisons were performed using repeated measures analysis with pairwise comparisons; Bonferroni corrections were applied to adjust for multiple comparisons. Additionally, data were reorganized to compare CHX (Peridex^®^) and SL (StellaLife^®^) groups directly using group statistics and *t*-tests, with Levene’s test employed to verify homogeneity of variances. All analyses were conducted using SPSS version 26 (IBM, Armonk, NY, USA), with statistical significance set at *p* < 0.05.

## 3. Results

### 3.1. Intergroup Comparisons of Clinical Periodontal Parameters

Baseline comparisons using independent *t*-tests confirmed that Groups A and B were statistically similar in all measured clinical parameters. Pairwise comparisons were performed at baseline, 2 weeks, and 4 weeks within each group. In both groups, significant improvements were observed in periodontal parameters from baseline to the 2-week and 4-week evaluations. Specifically, significant reductions in probing depth (PD), bleeding on probing (BOP), gingival margin (GM) alterations, clinical attachment loss (CAL), gingival index (GI), and plaque index (PI) were recorded. Small reductions in mean CAL values were recorded between baseline and follow-up visits; however, these changes were within the expected clinical measurement variability.

Moreover, in Group B, comparisons between week 2 and week 4 revealed significant further improvements in PD and GM (*p* < 0.05), indicating continued periodontal improvement over time.

### 3.2. Comparison of CHX Versus SL on Clinical Periodontal Findings

When clinical outcomes were directly compared between the CHX regimen and the SL regimen, mean values for PD, BOP, GM, CAL, PI, and GI were found to be comparable (Table 1). Statistical analysis using *t*-tests demonstrated no significant differences between the two treatment modalities for these parameters (Table 2; *p* > 0.05). Similarly, the mean gingival crevicular fluid (GCF) volume recorded for the CHX group was 39.03, while that for the SL group was 38.79, with no statistically significant difference between them (Table 3; *p* > 0.05).

### 3.3. Comparison of CHX Versus SL on Microbiological Findings

Microbiological assessment using PCR measured the genome copies per milliliter of key periodontal pathogens at baseline, after CHX and SL (Table 4) Using *t*-tests (with equal variances confirmed by Levene’s test), no significant differences were detected in the levels of these periodontal pathogens (Table 5) between the CHX and SL groups (*p* > 0.05).

### 3.4. Summary of Findings

**Figure 1:**
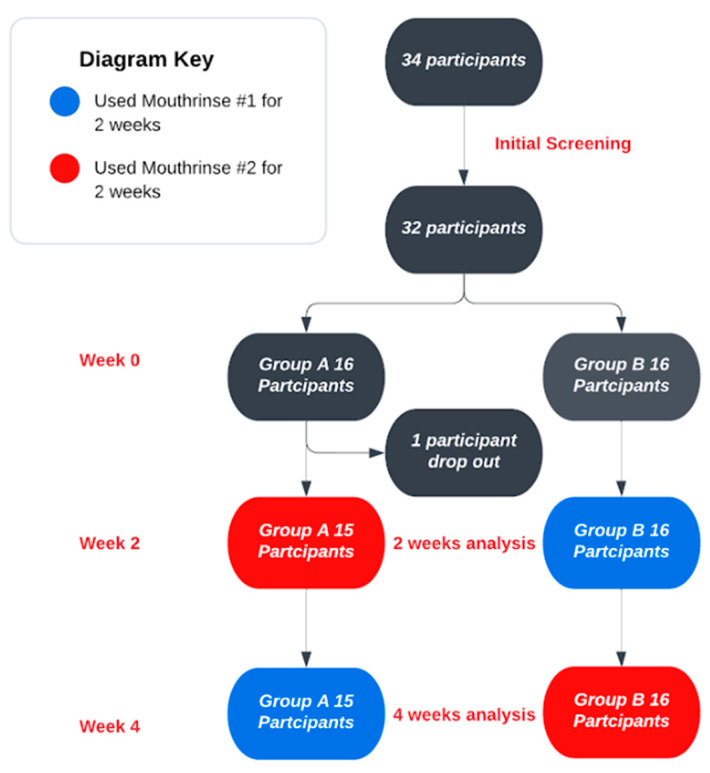
Clinical Trial Design Flowchart.

**Figure 2:**
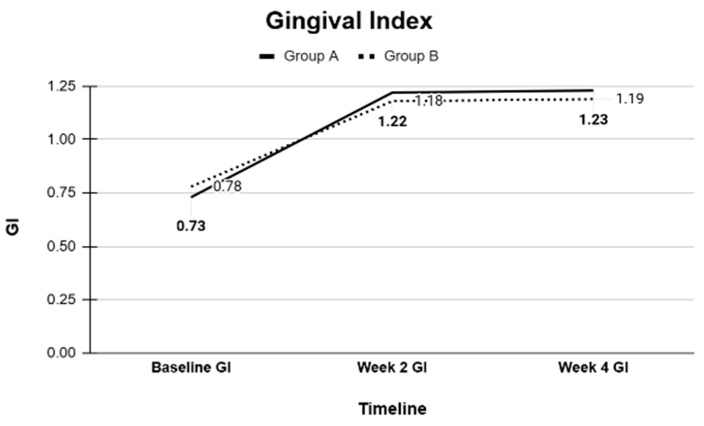
Gingival index at Baseline, Week 2, and Week 4 measured as an average of Group A and B as follows: 0: Normal gingiva; 1: Mild inflammation—no bleeding on probing; 2: Moderate inflammation—bleeding on probing; 3: Severe inflammation—spontaneous bleeding.

**Figure 3:**
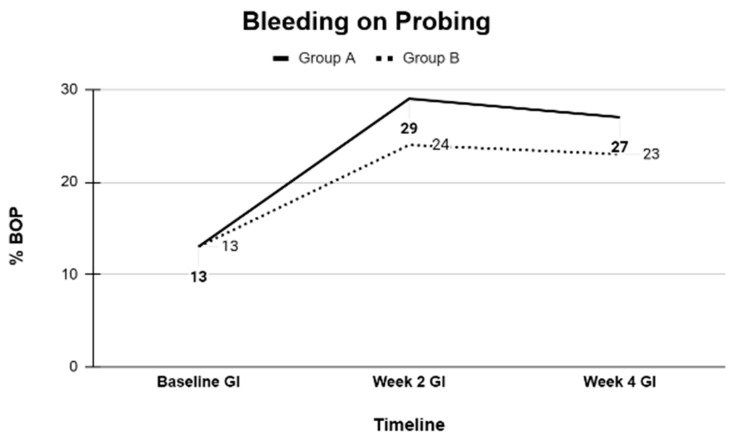
Number of dentitions with bleeding on probing at Baseline, Week 2, and Week 4 as an average of Group A and B.

**Figure 4:**
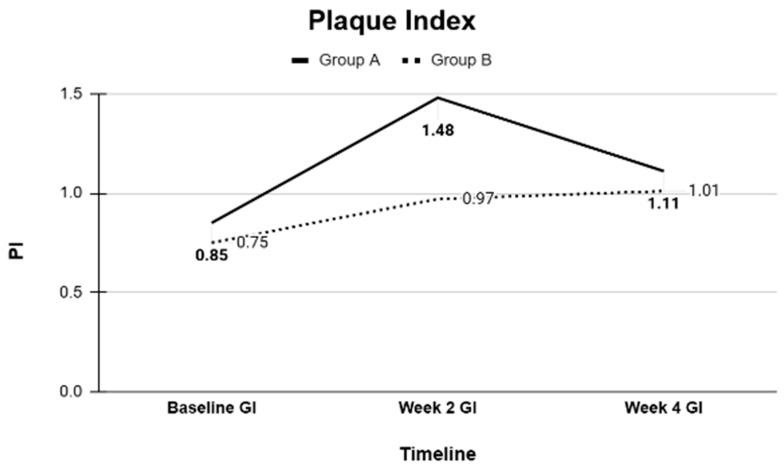
Plaque index at Baseline, Week 2, and Week 4 measured as an average of Group A and B as follows: 0: No plaque; 1: A film of plaque adhering to the free gingival; 2: Moderate accumulation of soft matter; 3: Abundance of soft matter.

**Figure 5:**
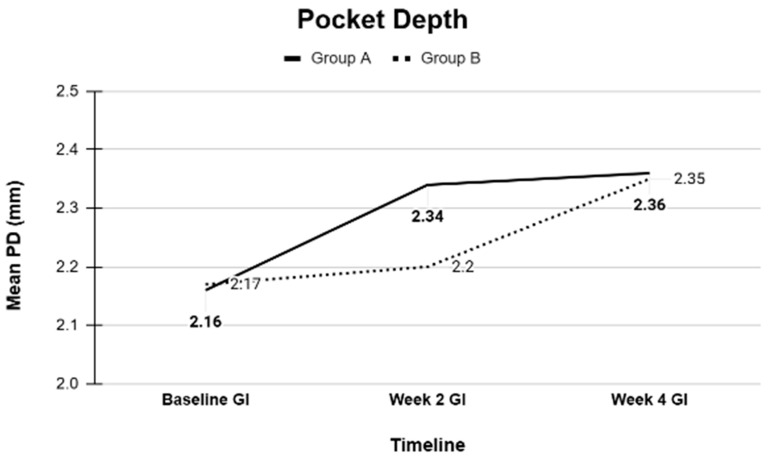
Pocket depth at Baseline, Week 2, and Week 4 measured as an average of all probing depths in Groups A and B.

Overall, the clinical periodontal parameters (PD, BOP, GM, CAL, GI, and PI) and GCF volumes, as well as the microbiological profiles of key periodontal bacteria, were comparable between the CHX and SL regimens (Table 1, Table 2, Table 3, Table 4 and Table 5). Both treatment modalities produced significant improvements from baseline, with no statistically significant differences between them at the study’s conclusion (Figure 1, Figure 2, Figure 3, Figure 4 and Figure 5).

## 4. Discussion

This study evaluated the clinical efficacy of SL, an herbal-based oral care product, compared to the conventional 0.12% chlorhexidine (CHX) regimen in an experimental gingivitis model among dental students. It should be noted that antiseptics are not typically required for routine management of gingivitis, which generally resolves within 7–10 days following professional prophylaxis and adequate oral hygiene. In this study, the experimental gingivitis model was used as a validated framework to compare the short-term effects of SL and CHX under standardized plaque accumulation conditions, rather than to suggest that antiseptics are necessary for standard gingivitis treatment.

Our findings revealed no statistically significant differences between the two treatment modalities across key periodontal parameters—including plaque index, gingival index, probing pocket depth, bleeding on probing, gingival margin, clinical attachment loss, and gingival crevicular fluid (GCF) volume.

It should be noted that gingivitis is not associated with clinical attachment loss; therefore, CAL values are generally expected to remain unchanged. The small variations in CAL observed in this study most likely reflect normal probing variability (±1 mm) and changes in gingival margin position due to resolution of inflammation, rather than true attachment gain. The only clinical parameter typically expected to improve in gingivitis is probing depth, as pseudo-pockets resolve with decreased gingival swelling.

These results suggest that SL, an herbal-based oral care product, may provide a level of clinical efficacy comparable to that of CHX in reducing plaque accumulation and gingival inflammation while also mitigating some of the adverse effects associated with CHX, such as staining and altered taste sensation. In addition, the bacterial reduction was comparable between the CHX and SL regimens.

The in vitro data from previous studies provided a strong rationale for our investigation. Studies by Fujioka-Kobayashi et al. [18] and Zhou et al. [19] demonstrated that SL not only preserved cell viability better than CHX but also promoted enhanced wound healing through increased fibroblast migration and collagen deposition. Although our clinical outcomes did not show statistically significant differences, the comparable efficacy observed in our study, combined with the superior in vitro biocompatibility of SL, suggests that this herbal formulation could offer additional advantages over CHX, particularly in terms of wound healing and patient comfort [9]. The enhanced biocompatibility of SL, as evidenced by previous in vitro studies showing increased fibroblast viability, accelerated wound closure, and substantially greater collagen deposition compared to CHX, suggests a safer, more patient-friendly alternative for post-surgical oral care [9,18,19]. Moreover, the additional benefits related to pain management and reduced reliance on opioids further position SL as a multifaceted therapeutic option that could improve overall patient comfort and outcomes in periodontal therapy [25,26]. However, patient comfort was not an outcome variable of this study, and current studies evaluating the pain management properties of SL are limited to oral surgery procedures, warranting future studies with patient-reported outcomes on SL in periodontal therapy [27].

Overall, the results of this study contribute to a growing body of evidence supporting the potential of herbal-based oral care products in periodontal therapy [13,27]. In a randomized controlled clinical trial comparing the effects of a neem-based mouth rinse to CHX in 54 patients, no significant differences were found in all clinical and microbiological parameters [27]. In another recent in vitro study by Tsai et al. [28]. SL inhibited the growth of *F. nucleatum* while showing lower toxicity toward commensal bacteria such as *S. oralis*, and *S. gordonii* when compared to CHX and Listerine. The selective cytotoxicity of SL appears to offer a promising alternative to traditional CHX rinses, combining effective antimicrobial action and improved wound healing properties [9]. Continued research in this area may lead to broader clinical adoption of herbal-based formulations, ultimately advancing patient care and improving long-term periodontal health.

While CHX has been associated with adverse effects such as altered taste, tooth staining, and detrimental effects on gingival fibroblasts, SL appears to maintain a more favorable environment for oral tissue repair [9]. In clinical settings, where tissue healing is paramount—such as post-surgical scenarios—this improved biocompatibility may translate into better long-term outcomes, even if short-term clinical parameters do not differ markedly between the two products. While post-operative surgery was outside the scope of this current study, the experimental gingivitis model may reflect what patients experience as they often refrain from and/or limit their oral hygiene practices 2 weeks post-surgery. Thus, clinicians may utilize this study to support their use of SL in post-surgery patients to minimize plaque scores and gingival inflammation.

One limitation of this study is that the relatively small sample size (34 participants) and short treatment duration may have limited the detection of subtle differences between the regimens. Although the cross-over design helped mitigate inter-individual variability, a larger sample would provide more statistical power and improve the generalizability of our findings. Additionally, reliance on self-reported adherence to the protocol (including restrictions on toothbrushing, flossing, and the timing of eating or drinking post-application) may introduce potential bias, and the homogeneous nature of the study population (dental students) may affect the generalizability and external validity of the findings. Dental students are likely more motivated and knowledgeable about oral hygiene practices than the general population, which could influence their compliance and overall treatment response. Future studies involving larger, more diverse populations and longer follow-up periods, along with objective compliance measures, are warranted to further validate the clinical benefits of SL.

Another potential limitation of this study is that the duration of each treatment phase was only two weeks. This relatively short observation period might not have been sufficient to capture longer-term differences in periodontal healing or microbiome (in biofilm) changes. Future studies with extended follow-up periods are warranted to evaluate the sustainability of the treatment effects and to assess any delayed benefits or adverse outcomes.

Another limitation of this study is the imbalance between the treatment regimens. The StellaLife protocol included both a mouth rinse and a gel containing active ingredients, whereas the chlorhexidine group received a rinse plus a placebo gel. This difference means that SL participants were exposed to two active formulations, while CHX participants had only one. Although this design reflected the commercially available SL system, it may have introduced bias in favor of SL, and future studies should compare formulations with equivalent modes of delivery to ensure balanced assessment.

Lastly, salivary samples were used for microbiological analysis instead of plaque samples. While saliva provides a global, reproducible overview of oral microbiota, it does not fully capture site-specific plaque biofilm composition. Future studies should incorporate subgingival plaque sampling to provide more detailed, site-level microbiological insights.

Moreover, only a limited number of periodontal pathogens were investigated in this study, which restricts the ability to evaluate broader microbiota changes or shifts in microbial diversity. Comprehensive microbiome analyses would provide a more complete understanding of the ecological impact of SL compared with CHX.

Under the tested conditions, SL demonstrated clinical efficacy comparable to CHX in managing experimental gingivitis; however, the CHX regimen achieved these outcomes with a single rinse application, whereas the SL regimen required both a rinse and a gel. The potential advantages of SL, particularly its enhanced biocompatibility and wound-healing properties demonstrated in preclinical studies, warrant further investigation in larger, more diverse populations and in longer-term clinical trials. Future research should also incorporate objective measures of patient compliance and explore patient-reported outcomes such as comfort, taste, and overall satisfaction with the treatment regimen.

In conclusion, this randomized, controlled, cross-over clinical trial demonstrated that the SL regimen produced clinical outcomes comparable to those of 0.12% CHX in the management of experimental gingivitis. Given that the SL regimen included both a rinse and an active gel, while CHX was used as a rinse alone, these results should be interpreted with caution. The comparable efficacy observed suggests that CHX may represent a more efficient approach under the tested conditions, while SL warrants further study in formulations, allowing balanced comparison. Certainly, the negative effects of CHX, such as taste alterations, staining of teeth and restorations, as well as the content of alcohol, should be considered in CHX applications.

Within the limitations of the short, two-week intervention period and the homogenous student population, these findings should be interpreted with caution. SL may represent a promising adjunctive option, but further long-term studies in more diverse populations are necessary to confirm its efficacy, safety, and broader clinical applicability.

## Figures and Tables

**Table 1:** Comparison of plaque index, gingival index, pocket depths, bleeding on probing, gingival margin and clinical attachment levels between the experimental groups of Chlorhexidine and StellaLife^®^.

	Group	N	Mean	Std. Deviation	Std. Error Mean
**PI**	**CHX**	31	1.04	0.39	0.07
	**SL**	31	1.24	0.43	0.08
**GI**	**CHX**	31	1.21	0.19	0.03
	**SL**	31	1.20	0.19	0.03
**PD**	**CHX**	31	2.28	0.17	0.03
	**SL**	31	2.34	0.15	0.03
**BOP (%)**	**CHX**	31	25.47	13.53	2.43
	**SL**	31	25.93	13.65	2.45
**GM**	**CHX**	31	−0.42	0.16	0.03
**CAL**	**SL**	31	−0.43	0.14	0.02
	**CHX**	31	1.86	0.13	0.02
	**SL**	31	1.92	0.13	0.02

**Table 2:** No statistically significant difference was found between the groups for clinical parameters including plaque index, gingival index, pocket depths, bleeding on probing, gingival margin and clinical attachment levels.

Levene’s Test for Equality of Variances					
	F	Sig.	t	df	Sig. (2-Tailed)
**PI**	1.843	0.180	−1.944	60	0.057
**GI**	0.028	0.868	0.050	60	0.960
**PD**	0.164	0.687	−1.613	60	0.112
**BOP %**	0.043	0.837	−0.133	60	0.895
**GM**	0.726	0.397	0.296	60	0.768
**CAL**	0.539	0.466	−1.614	60	0.112

**Table 3:** Periotron values comparing chlorhexidine and StellaLife^®^ with no statistically significant difference in the values.

Group Statistics Periotron					
	N	Mean	Std. Deviation	Std. Error Mean	Sig. (2-Tailed)
**CHX**	62	39.03	21.396	2.717	0.951
**SL**	62	38.79	22.774	2.892	0.951

**Table 4:** Mean bacterial count at baseline, CHX use and SL use.

Bacteria	Group	N	Mean (Bacterial Count)	Std. Deviation
***A. actinomycetemcomitans***	BASE	31	3.25 × 10^5^	1.78 × 10^6^
	CHX	31	4.03 × 10^4^	1.30 × 10^5^
	SL	31	7.63 × 10^4^	3.78 × 10^5^
***P. gingivalis***	BASE	31	4.58 × 10^4^	1.28 × 10^5^
	CHX	31	1.29 × 10^4^	5.92 × 10^4^
	SL	31	1.08 × 10^5^	5.38 × 10^5^
***T. denticola***	BASE		9.44 × 10^4^	2.74 × 10^5^
	CHX	31	6.93 × 10^4^	2.38 × 10^5^
	SL	31	7.16 × 10^4^	1.63 × 10^5^
***T. forsythia***	BASE	31	3.13 × 10^5^	6.77 × 10^5^
	CHX	31	2.99 × 10^5^	8.87 × 10^5^
	SL	31	3.04 × 10^5^	6.92 × 10^5^
***F. nucleatum***	BASE	31	1.49 × 10^7^	4.04 × 10^7^
	CHX	31	1.58 × 10^7^	4.29 × 10^7^

**Table 5:** Statistical comparison of the periodontopathogenic bacteria within the two experimental groups showing no statistically significant differences.

Bacteria	F	Sig.	t	df	Sig. (2-Tailed)
*** 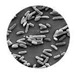 *** ***A. actinomycetemcomitans***	1.019	0.317	−0.502	60	**0.618**
*** 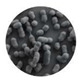 *** ***P. gingivalis***	3.560	0.064	−0.975	60	**0.333**
*** 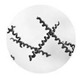 *** ***T. denticola***	0.001	0.971	−0.045	60	**0.964**
*** 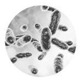 *** ***T. forsythia***	0.022	0.882	−0.027	60	**0.978**
*** 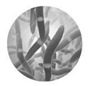 *** ***F. nucleatum***	0.139	0.711	−0.260	60	**0.796**

## Data Availability

The data that support the findings of this study are not publicly available due to participant confidentiality but are available from the corresponding author upon reasonable request and with appropriate institutional and ethical approval.

## References

[1] Van der Weijden G., Hioe K. (2005). A systematic review of the effectiveness of self-performed mechanical plaque removal in adults with gingivitis using a manual toothbrush. J. Clin. Periodontol..

[2] Kinane D.F., Stathopoulou P.G., Papapanou P.N. (2017). Periodontal diseases. Nat. Rev. Dis. Primers.

[3] Listgarten M.A. (1988). The role of dental plaque in gingivitis and periodontitis. J. Clin. Periodontol..

[4] Rosan B., Lamont R.J. (2000). Dental plaque formation. Microbes Infect..

[5] Ahmad P., Escalante-Herrera A., Marin L.M., Siqueira W.L. (2025). Progression from healthy periodontium to gingivitis and periodontitis: Insights from bioinformatics-driven proteomics—A systematic review with meta-analysis. J. Periodontal Res..

[6] Reddy M.S., Geurs N.C., Jeffcoat R.L., Proskin H., Jeffcoat M.K. (2000). Periodontal disease progression. J. Periodontol..

[7] Greenstein G., Berman C., Jaffin R. (1986). Chlorhexidine: An adjunct to periodontal therapy. J. Periodontol..

[8] Fiorillo L., D’Amico C., Mehta V., Cicciù M., Cervino G. (2024). Chlorhexidine cytotoxicity on oral Behaviors: Last 20 Years systematic review. Oral Oncol. Rep..

[9] Estrin N.E., Romanos G.E., Tatch W., Pikos M., Miron R.J. (2022). Biological Characterization, Properties, and Clinical Use of a Novel Homeopathic Antiseptic Oral Recovery Kit: A Narrative Review. Oral Health Prev. Dent..

[10] Mueller M., Hobiger S., Jungbauer A. (2010). Anti-inflammatory activity of extracts from fruits, herbs and spices. Food Chem..

[11] Gupta S.C., Prasad S., Tyagi A.K., Kunnumakkara A.B., Aggarwal B.B. (2017). Neem (*Azadirachta indica*): An indian traditional panacea with modern molecular basis. Phytomedicine.

[12] Hao F., Kumar S., Yadav N., Chandra D. (2014). Neem components as potential agents for cancer prevention and treatment. Biochim. Biophys. Acta (BBA)-Rev. Cancer.

[13] Elavarasu S., Abinaya P., Elanchezhiyan S. (2012). Evaluation of anti-plaque microbial activity of *Azadirachta indica* (neem oil) in vitro: A pilot study. J. Pharm. Bioallied Sci..

[14] Biswas K., Chattopadhyay I., Banerjee R.K., Bandyopadhyay U. (2002). Biological activities and medicinal properties of neem (*Azadirachta indica*). Curr. Sci..

[15] Chakraborthy G. (2008). Antimicrobial activity of the leaf extracts of *Calendula officinalis* (Linn). J. Herb. Med. Toxicol..

[16] Chiang L.-C., Chiang W., Chang M.-Y., Lin C.-C. (2003). In vitro cytotoxic, antiviral and immunomodulatory effects of Plantago major and Plantago asiatica. Am. J. Chin. Med..

[17] Khairnar M., Pawar B., Marawar P., Mani A. (2013). Evaluation of Calendula officinalis as an anti-plaque and anti-gingivitis agent. J. Indian Soc. Periodontol..

[18] Fujioka-Kobayashi M., Schaller B., Pikos M.A., Sculean A., Miron R.J. (2020). Cytotoxicity and gene expression changes of a novel homeopathic antiseptic oral rinse in comparison to chlorhexidine in gingival fibroblasts. Materials.

[19] Zhou P., Chrepa V., Karoussis I., Pikos M.A., Kotsakis G.A. (2021). Cytocompatibility Properties of an Herbal Compound Solution Support In Vitro Wound Healing. Front. Physiol..

[20] Socransky S.S., Haffajee A.D., Cugini M.A., Smith C., Kent R.L. (1998). Microbial complexes in subgingival plaque. J. Clin. Periodontol..

[21] Hajishengallis G. (2014). The inflammophilic character of the periodontitis-associated microbiota. Mol. Oral Microbiol..

[22] Löe H., Silness J. (1963). Periodontal disease in pregnancy I. Prevalence and severity. Acta Odontol. Scand..

[23] Silness J., Löe H. (1964). Periodontal disease in pregnancy II. Correlation between oral hygiene and periodontal condition. Acta Odontol. Scand..

[24] Cohen J. (1992). A power primer. Psychol. Bull..

[25] Tatch W. (2019). Opioid prescribing can be reduced in oral and maxillofacial surgery Practice. J. Oral Maxillofac. Surg..

[26] Lee C., Suzuki J.B. (2019). The efficacy of preemptive analgesia using a non-opioid alternative therapy regimen on postoperative analgesia following block bone graft surgery of the mandible: A prospective pilot study in pain management in response to the opioid epidemic. Clin. J. Pharmacol. Pharmacother..

[27] Botelho M.A., dos Santos R.A., Martins J.G., Carvalho C.O., Paz M.C., Azenha C., Ruela R.S., Queiroz D.B., Ruela W.S., Marinho G. (2008). Efficacy of a mouthrinse based on leaves of the neem tree (*Azadirachta indica*) in the treatment of patients with chronic gingivitis: A double-blind, randomized, controlled trial. J. Med. Plants Res..

[28] Tsai Y.-W.C., Siddiqui D.A., Kotsakis G.A. (2023). Selective antimicrobial effects of an herbal compound rinse against multi-species oral biofilms. Res. Sq..

